# Social Games: The Intersection of Established Reward Structures and Exercise for Older Rural Adults

**DOI:** 10.1093/geroni/igaa057.2090

**Published:** 2020-12-16

**Authors:** Lyn Holley

**Affiliations:** University of Nebraska Omaha, Omaha, Nebraska, United States

## Abstract

A high percentage of older adults engages with games; however research about impacts of gaming on older adults is focused almost exclusively on games intended to strengthen cognitive health or slow age-related cognitive decline, very little relates gaming to exercise. Rural –dwelling older adults are underrepresented in this as in other health-related research. Nonetheless, recent developments that explore effectiveness of embedding positive health behaviors (including exercise) in established games such as Bingo hold great promise for addressing the endemic social isolation and chronically debilitating conditions often associated with rural aging. Using recent research with a challenging population group (rural Appalachian older adults) as an anchor for discussion, this paper describes the state of the art of such interventions and, through a multinational lens, finds that these suggest a new direction for gaming design that could enhance effectiveness of the use of gaming to improve health and happiness of rural adults. Part of a symposium sponsored by the Rural Aging Interest Group.

